# Analyzing multidimensional formal dynamic models in psychology: A tutorial using graphical tools

**DOI:** 10.3758/s13428-026-03077-y

**Published:** 2026-06-22

**Authors:** Jingmeng Cui, Dieta Wagenmakers, G. Sander van Doorn, Fred Hasselman, Anna Lichtwarck-Aschoff

**Affiliations:** 1 Faculty of Behavioural and Social Sciences, University of Groningen, Groningen, the Netherlands; 2 Research Group of Quantitative Psychology and Individual Differences, KU Leuven, Tiensestraat 102, 3000 Leuven, Belgium; 3 Groningen Institute for Evolutionary Life Sciences, University of Groningen, Groningen, the Netherlands; 4 Behavioural Science Institute, Radboud University, Nijmegen, the Netherlands

**Keywords:** Formal dynamic models, Dynamic systems, Differential equations, Stability analysis, Bifurcation analysis

## Abstract

Formal theories translate verbal theories into a mathematical representation, such as a coupled differential equation or other dynamical systems, intending to strengthen the deductive power of (clinical) theories and to formulate testable and novel hypotheses. Work in clinical formal theories mainly relies on simulations, which is an intuitive method for evaluating overall model performance, but may fall short of establishing a precise link between the mathematical properties of the model and the dynamic properties of its outcome. Moreover, when the model’s outcome contradicts clinical observations, it is unclear where the discrepancy lies and how to improve the model. In this article, we introduce formal mathematical techniques for graphical model analysis, including phase plane analysis, which allows identifying a system’s stable and unstable equilibria, and bifurcation analysis, a framework to delineate parameter regimes corresponding to qualitatively different dynamical outcomes for a model. Using two formal dynamic models in psychology (one for panic disorder and one for suicidal ideation), we illustrate those methods through an easy-to-use R package, *deBif*, with a graphical user interface. These examples demonstrate the importance of using graphical tools to investigate the hypothesized mechanisms of psychological systems.

**Supplementary Information:**

The online version contains supplementary material available at 10.3758/s13428-026-03077-y.

## Introduction

Psychology has a long history of theory development, yet the integration and examination of theories still face significant obstacles (cf. Meehl, 1990a, 1990b). Many scholars have used the term “theory crisis” to describe the fact that many theories appear to coexist in many fields of psychology, even though they often seek to explain the same empirical phenomena. They almost all lack the ability to generate predictions that would allow experimentalists to distinguish between the veracity of competing theories (i.e., by comparing their empirical accuracy), and they lack the required level of formal description that would allow theorists to determine whether they should be amended, integrated with competing theories, or have to be abandoned entirely (Eronen & Bringmann, 2021; Fried, 2020; Oberauer & Lewandowsky, 2019). One possible avenue to advance theory construction that has been proposed by many researchers is the use of formal dynamic models as a way to explicate psychological theories, which should allow for the deduction of more precise testable predictions (Borgstede & Eggert, 2023; Borsboom et al., 2021; Haslbeck et al., 2022; Robinaugh et al., 2021, 2024, but also see Eronen & Bringmann, 2021; Oude Maatman, 2025, for opposite opinions). By using formal models, researchers can translate verbal theories about psychological phenomena into mathematical representations of how psychological processes in an individual system interact and evolve. Some early formal models in psychology include the classical conditioning model by Rescorla and Wagner (1972), the hand movement model by Haken et al. (1985), and the cognitive development model by van Geert (1991).

One type of formal model that was recently proposed is the formal dynamic model defined using ordinary differential equations (ODEs) or stochastic differential equations (SDEs). A distinct feature of these models is their ability to simulate how psychological processes unfold over time, allowing researchers to compare them to the real-life psychological phenomena (Borsboom et al., 2021; Robinaugh et al., 2021). Many formal dynamic models have been proposed in the past years (Burger et al., 2020; Schöller et al., 2018; van Dongen et al., 2025; Wang et al., 2023), and some researchers have also advocated using formal dynamic models to integrate interpersonal and intrapersonal theories (Borsboom & Haslbeck, 2024) and test the effectiveness of psychotherapies *in silico* (Ryan et al., 2025).

Most recent studies on formal dynamic models rely solely on simulations to generate predictions and evaluate models. Usually, simulation results are used to either qualitatively compare them to the phenomena observed in real life (Burger et al., 2020; Robinaugh et al., 2024; Schöller et al., 2018) or make comparisons between some statistical indicators of the simulated data and real-life data (Haslbeck et al., 2022). An ultimate validation of the formal model would be the case in which an as-yet-unknown phenomenon is predicted, which is subsequently corroborated to exist in an empirical study (e.g., Simmering et al., 2008; Spencer et al., 2001). Simulation-based approaches are intuitive and easily built on the existing toolkits of psychologists, yet they also have considerable shortcomings. A key limitation is that simulations do not provide direct insight into what the critical components and interactions are that give rise to a certain phenomenon (Cui et al., 2023). When the simulation output is in accordance with real-life observations, we do not know whether this only holds for a very specific set of function forms and parameter values, or whether it also generalizes to a wider range of (similar) conditions. When the simulation output is not in line with the real-world phenomenon, we do not know what the problem is, nor what we can do to solve it. Those shortcomings may limit progress in studying the potential of formal dynamic models in advancing psychological theories.

Several other fields of science, such as biology and physics, have a much longer history of applying dynamic models to advance theories, and they have developed concrete mathematical methods for analyzing them that we can also apply in psychology. One helpful technique, for example, is the *phase plane analysis* (Kuznetsov, 2023). This technique uses both mathematical calculations and graphical representations to determine the system's equilibria and to study its evolution from a given starting point. Another technique is the *bifurcation analysis* (Kuznetsov, 2023), which focuses on the parameter space instead of the variable space. By using this technique, we can systematically investigate how the parameter setting changes the stability features of the system and explore the qualitatively different patterns of the system.

Although earlier work in psychology (especially bivariate models in dyadic interactions, e.g., Gottman et al., 2002; Strogatz, 1988; also see van der Maas, 2024) often applies phase plane analysis and bifurcation analysis, it is not the case for more recent and complicated models, primarily in psychopathology. Only a few of them have applied a simple version of phase plane analysis (Cui et al., 2023; Cui, Olthof, et al., 2025b, 2025a; Dablander et al., 2023; Robinaugh et al., 2024), whereas bifurcation analysis has been used even less (but see Verdonck & Tuerlinckx, 2014, for a notable use case). On the other hand, although many excellent textbooks on phase plane analysis and bifurcation analysis exist in various fields (Jordan & Smith, 2007; Kuznetsov, 2023; also see a recent textbook in the psychology field by van der Maas, 2024), they are mostly built on classical dynamic systems models in mathematics, ecology, and biology. Hands-on tutorial materials on psychological formal models, especially their growing applications in psychopathology involving multiple variables, remain scarce, potentially hindering their broader adoption in psychology.

Therefore, in this tutorial, we aim to provide a comprehensive introduction to both techniques and illustrate their usage with concrete examples in psychological contexts. The graphical tools that we introduce here should not be seen as replacements for the current simulation-based approach. Rather, we see those different approaches as complementary: the graphical tools are important for understanding the role of elements and interactions in a deterministic setting, whereas a simulation-based approach can better accompany situations with noise and when there is a rather large number of interacting elements involved. The intended readership of the manuscript is the researchers with applied backgrounds, interested in applying formal dynamic models for theory building, but less familiar with the specific tools and software. For the researchers with a deeper mathematical background, the textbooks by Jordan and Smith (2007), Kuznetsov (2023), Strogatz (2018), and van der Maas (2024) may provide more technical details. To improve the readability of the manuscript, we limit the amount of mathematical derivations and provide graphical tools to reduce potential barriers to applying them to psychological systems. The tutorial is structured as follows. First, we introduce the basic ideas of formal dynamic models in psychology and outline two example models. Second, we explain the two techniques, namely the phase plane analysis and the bifurcation analysis, by analyzing these two models. Third, we illustrate the usage of those two techniques by applying them to the two example systems. Finally, we discuss the role of the analyses in modeling and reflect on the purpose of modeling and the limitations of such approaches.

## Introduction of formal dynamic models in psychology

**Fig. 1:**
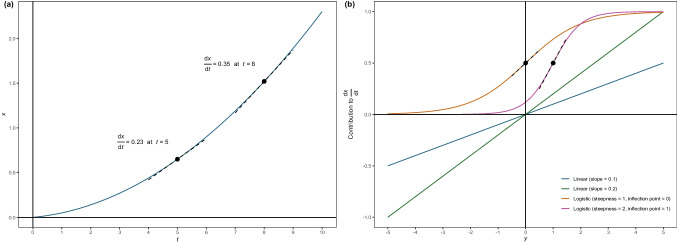
Illustrations of **(a)** changing rate and **(b)** meaning of parameters in linear and logistic functions. *Reference lines* at zero are shown on both axes for clarity

A formal dynamic model describes how the state of a system evolves over time, using formal, mathematical language. Specifically, most models use a mathematical formula to specify the *rate of change* of a variable. In mathematics, this rate of change is represented by the *time derivative*, $\mathrm{d}x/\mathrm{d}t$. It corresponds to the slope of the tangent line on the time series plot (Fig. 1a). If the rate of change is positive, the variable will increase over time; if it is negative, it will decrease. For example, if we use *x* to represent a person’s level of happiness, and $\mathrm{d}x/\mathrm{d}t$ is positive, we know that the person is becoming *happier* for the coming period of time.

The rate of change of a variable may also change over time, and may be influenced by the value of itself but also by other variables. For a psychological system, we are often interested in how one variable contributes to the change in itself or another variable. For example, when a person’s level of happiness is high, it may tend to decrease and go back to the normal level; when a person has social interactions with friends, the person’s level of happiness tends to increase. If we use *x* to represent the person’s level of happiness, and use *y* to represent the person’s frequency of interaction with friends, we may write the dynamic equation of *x* as follows:

1 $$\frac{\mathrm{d}x}{\mathrm{d}t}=ay-bx,$$

where *a* and *b* are *parameters* of the model. We can then alter the parameters to change the strength of the influences. For example, if we specify a=0.2, it means that the strength of the influence from social interaction on happiness is higher than when we specify a=0.1. For a linear term, the meaning of the parameter is relatively clear, as it represents the slope of a linear function (see Fig. 1b, linear functions). However, the function may also take a more complex, nonlinear form. A nonlinear function that is frequently used is the logistic function, which takes the form of:

2 $$f\left(y\right)=\frac{1}{1+\mathrm{exp}\left(-k\left(y-h\right)\right)},$$

where *h* represents the *inflection point*, where the function changes from curving upward to curving downward, and *k* represents the *steepness* of the function, which is proportional to the slope at the inflection point (see Fig. 1b, logistic functions). If we assume the influence of *y* on *x* takes a nonlinear logistic form, we can rewrite Equation 2 as follows:

3 $$\frac{\mathrm{d}x}{\mathrm{d}t}=\frac{1}{1+\mathrm{exp}\left(-k\left(y-h\right)\right)}-bx.$$

When there are multiple variables, for simplicity, we often use a vector, denoted by a letter in bold (e.g., ${\boldsymbol{x}}$), to represent all of them collectively. This allows us to represent $\mathrm{d}x/\mathrm{d}t$, $\mathrm{d}y/\mathrm{d}t$, etc., with the following compact form:

4 $$\frac{\mathrm{d}{\boldsymbol{x}}}{\mathrm{d}t}={\boldsymbol{f}}\left({\boldsymbol{x}}\right).$$

Such equations are called *ordinary differential equations* (ODEs), which form the basis of most formal models in psychology. Often, the model also includes random fluctuations. The ODEs then become *stochastic differential equations* (SDEs), which take the general form:

5 $$\mathrm{d}{\boldsymbol{x}}={\boldsymbol{f}}\left({\boldsymbol{x}}\right)\mathrm{d}t+{\boldsymbol{g}}\left({\boldsymbol{x}}\right)\mathrm{d}{\boldsymbol{W}},$$

where ${\boldsymbol{x}}$ represents all relevant variables, ${\boldsymbol{f}}({\boldsymbol{x}})$ represents the deterministic part of the changing rate of the system, and ${\boldsymbol{g}}({\boldsymbol{x}})$ represents the stochastic part of the system. Analyzing SDEs is relatively difficult, but analyzing the deterministic part of them (i.e., the ODEs) is often much easier. Therefore, we first focus on the ODE skeleton of the model.

As already shown in previous examples, the variables in psychological models may be emotions, perceptions, physiological states, or any other quantities that are deemed important for a certain research question (but also see discussions by Kalis & Borsboom, 2020; Oude Maatman, 2020). We now introduce two concrete formal dynamic models, which will be used as examples along the tutorial.

The first model is the panic disorder model by Robinaugh et al. (2024). The core variables in the model are perceived threat (*PT*) and physical arousal (*A*). According to previous theories by Clark (1986), the two variables can strengthen each other: perceived threat increases when physical arousal is higher, and vice versa, which forms a vicious circle, potentially leading to panic attacks. The theory does not specify the exact form of the function. Therefore, various types of functions may apply. In Robinaugh et al. (2024), the authors added another variable, homeostatic feedback (*H*), to make sure the system exhibits expected behavior. This variable *H* represents the strength of the body’s regulatory process that drives the arousal level back to the equilibrium level after a perturbation. The core part of this panic disorder model is as follows:

6 $$\frac{\mathrm{d}A}{dt}={r}_{A}\left({S}_{PT,A}PT-A-H\right),$$

7 $$\frac{\mathrm{d}PT}{\mathrm{d}t}={r}_{PT}\left(\frac{1}{1+\mathrm{exp}\left(-{k}_{PT}\left(A-{h}_{A,PT}\right)\right)}-PT\right),$$

8 $$\frac{\mathrm{d}H}{\mathrm{d}t}={r}_{H}\left(\frac{1}{1+\mathrm{exp}\left(-{k}_{H}\left(A-{h}_{A,H}\right)\right)}-0.5H\right).$$

**Fig. 2:**
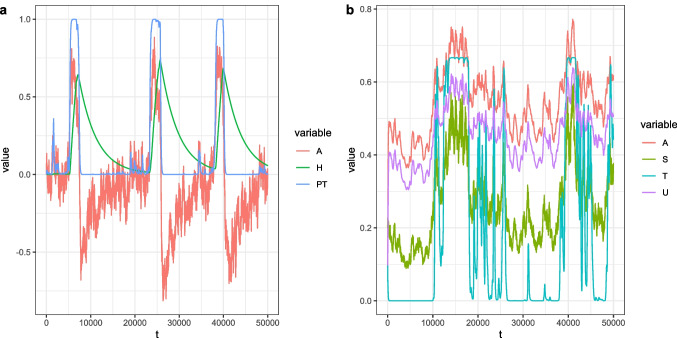
Simulation examples for **a** the panic disorder model and **b** the suicidal ideation model. We used the default parameter values shown in Tables 1 and 2 for the simulations. Random noise was added to specific variables according to the original specifications. For the panic disorder model, Gaussian noise with *sd* = 0.01 was added to *A*. This is a simpler form of noise than the one used by Robinaugh et al. (2024), as the form of noise is not the central topic of investigation of the current work, and we found that using this simpler form of noise in this model produced similar outputs. In addition, we removed the range restriction in the original model to avoid artificial influence on the mathematical property of the dynamic system. For the suicidal ideation model, we used the same form of geometric Brownian noise as used by Wang et al. (2023)

From the equations above, we can see that the rate of change of each variable is influenced by the value of itself and other variables. For example, in Eq. 6, *PT* has a positive influence on *A*, and *A* and *H* have negative influences on *A*. From the other direction (Eq. 7), *A* also has a positive influence on *PT*, but this function has a different form. The function of influence from *PT* to *A* is a straight line (Eq. 6), yet the function of influence from *A* to *PT* is an S-shaped logistic curve (Eq. 7; also see Fig. 1b). In a related article, the same group of authors (Robinaugh et al., 2021) tried various function forms and found that the combination shown in Eqs. 6–8 was the only one that produced the expected panic attacks. We will perform a detailed analysis of this conclusion in subsequent sections. In addition to the ones above, the original model by Robinaugh et al. (2024) also involves many other variables. We here focus on these three variables for simplicity, as only the interaction between these three variables can already produce the core phenomenon of panic attacks. A simulation example using default parameters (Table 1) is shown in Fig. 2a. The spikes shown in this figure represent panic attacks, characterized by short periods of high physical arousal (*A*) and perceived threat (*PT*).

**Table 1:** The default parameter values for the panic disorder model

Parameter	Interpretation	Value
${r}_{A}$	Overall rate of change of *A.*	0.5
${S}_{PT,A}$	Strength of the influence from *PT* on *A.*	1
${r}_{PT}$	Overall rate of change of *PT.*	1
S	Arousal schema, representing how much the person believes physical arousal is a sign of threat. It can alter the value of both ${k}_{PT}$ and ${h}_{A,PT}$.	1
${k}_{PT}$	Steepness (see Fig. 1b) of the rate of change of *PT*.	$20-10*{0.1}^{S}$
${h}_{A,PT}$	Inflection point (see Fig. 1b) of *A* for its influence on *PT.*	${0.25}^{S}$
${r}_{H}$	Overall rate of change of *H.*	0.05
${k}_{H}$	Steepness (see Fig. 1b) of the rate of change of *H*.	20
${h}_{A,H}$	Inflection point (see Fig. 1b) of *A* for its influence on *H.*	0.4

All model parameters are taken from the PanicModel package associated with Robinaugh et al. (2024). The package can be accessed from https://github.com/jmbh/PanicModel/. The original article did not specify the parameter ranges; however, to remain theoretically valid, all parameters should have positive values. The notations of the parameters by Robinaugh et al. (2024) are slightly different for the main text and for the R code. Here, we use the notation from the R code in the PanicModel package to ensure it aligns with the R code we provide in this manuscript

The panic disorder model has been used for illustration in many previous articles (e.g., Cui et al., 2023; Haslbeck et al., 2022; Robinaugh et al., 2021). To show the broad applicability of the tools introduced here, we also include another example, namely the model of suicidal ideation by Wang et al. (2023)^1^. In this model, the authors aim to explain the observation that suicidal thoughts often have a rapid onset, a short duration, and are close to zero most of the time, although the level of external stressors and aversive internal states may fluctuate a lot. The core variables in the model are aversive internal states (*A*), urge to escape (*U*), and suicidal thoughts (*T*). Their relationships are mathematically expressed as follows:

9 $$\frac{\mathrm{d}A}{\mathrm{d}t}={b}_{2}A\left(K-A\right)+{a}_{2}S-{d}_{2}T,$$

10 $$\frac{\mathrm{d}U}{\mathrm{d}t}={-c}_{3}U+{b}_{3}A,$$

11 $$\frac{\mathrm{d}T}{\mathrm{d}t}=-{d}_{4}T+\frac{1}{1+\mathrm{exp}\left[-{c}_{41}\left(U-{c}_{42}\right)\right]},$$

where *S* represents stressors, and other letters represent model parameters. The default parameter values of this model are summarized in Table 2, and a simulation example of the variables using the default parameters is shown in Fig. 2b.

**Table 2:** The default parameter values for the suicidal ideation model

Parameter	Interpretation	Value
${a}_{2}$	Strength of influence from *S* to *A*	1.5
${b}_{2}$	Self-regulating effect of *U*	1.5
${d}_{2}$	Strength of influence from *T* to *A*	0.1
K	The carrying capacity of *A*. If *A* is close to this value, it tends to converge toward it	0.1
${b}_{3}$	Strength of influence from *A* to *U*	2.5
${c}_{3}$	Self-regulating effect of *U*	3
${d}_{4}$	Self-regulating effect of *T*	1.5
${c}_{41}$	Steepness (see Fig. 1b) of the rate of change of *T*	90
${c}_{42}$	Inflection point (see Fig. 1b) of *U* for its influence on *T*	0.5

All model parameters are taken from the code associated with Wang et al. (2023). The code can be accessed from https://github.com/ShirleyBWang/math_model_suicide. The original article did not state the range of the parameters, yet to remain theoretically valid, all the parameters should have positive values

Now we will move to the next section, in which we introduce the basic concepts of the two graphical tools.

## Two graphical tools

**Fig. 3:**
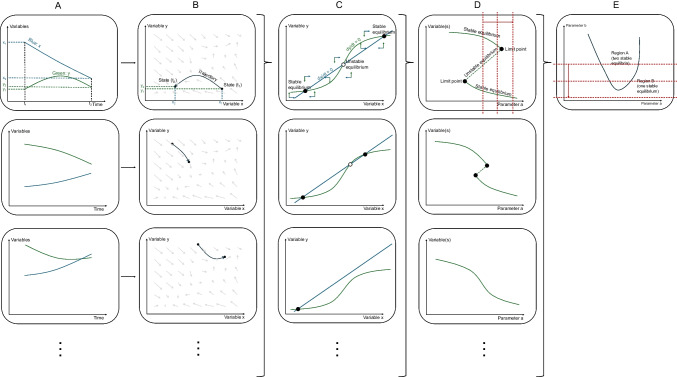
Illustration of several steps of the phase plane analysis and the bifurcation analysis. Column A: simulated time series of variables *x* and *y*. Column B: various trajectories on the phase plane of variables *x* and *y*; *gray arrows* in the background represent the vector field. Column C: phase plane analysis with nullclines and equilibrium points. Analysis results with various parameter values of *a* are shown. Column D: one-parameter bifurcation analysis of the parameter *a*. Three *red dashed lines* in the first plot correspond to the three plots in Column C. Only the values of variable *y* are shown for simplicity, while in the actual analysis, the values of multiple variables can be shown together. Analysis results with various parameter values of *b* are shown. Column E: two-parameter bifurcation analysis of both parameters *a* and *b*. Three *red dashed lines* in the first plot correspond to the three plots in Column D. The *vertical ellipses* under each column represent more possible plots that can be made under different parameter settings, and the *curly brackets* between columns mean that all the plots in the previous column can be summarized by one plot in the next column (e.g., all the trajectories in Column B can be summarized by the first plot in Column C)

In this section, we introduce important concepts used in two graphical tools for the analysis of the dynamics of formal models. We introduce an easy-to-use R package with a graphical interface that enables automated analysis of more complex systems. A brief illustration of the tools is shown in Fig. 3. As we can already see there, the graphical tools are shown in 2D spaces, which means that we can only analyze two variables or parameters at the same time. This does not mean that those methods can only be applied to dynamic systems with very few elements. As we will show later in concrete examples, we can use those graphical tools to analyze various parts of the system step-by-step and eventually reach a more comprehensive understanding of the dynamic features of the system as a whole.

The first graphical method is *phase plane analysis*. Here, the term phase plane can be understood as a two-dimensional plot^2^, in which each axis represents the value of a variable. Each point on the plot reflects a specific state of the system, characterized by the combined values of two variables, and a trajectory of the system can be shown as a line on the phase space (Fig. 3, Column B). A *vector field* can also be shown to represent the changing tendency from each starting point (Fig. 3, Column B). Compared to the time series plots (Column A), the phase plane plots represent information about variables while omitting time. Phase plane analysis aims to understand the direction of change, or how the state of the system evolves over time. For example, we can investigate in which range the variable of a system will increase, and when it will decrease (Column C). This is important for many psychological questions. If we know how to increase a person’s positive emotion, we can understand how to help the person escape the depressed state; if we know how to make the perceived threat decrease, we can understand when a person having a panic attack can gradually recover to the normal state. In order to know when a variable increases or decreases, we first need to find out the *nullcline*, the line on which the variable keeps the same value. The nullclines also set the boundaries of the increase and decrease regions. For example, in Column C, the blue and the green curves are nullclines for the two variables, *x* and *y*, respectively. On one side of the blue line, *x* increases, and on the other side, *x* decreases. We need to check the changing tendency on both sides to know which side corresponds to the increasing region. In our example, for the region above the blue curve, *x* increases, and for the region below the blue curve, *x* decreases. Similarly, we can check the changing tendency on both sides of the green line and infer that, for the region above the green curve, *y* decreases, and for the region below the green curve, *y* increases. At the intersection points, both variables are kept constant. Hence, those points are the *equilibrium points* of the system (Column C)*.* An equilibrium point can be stable or unstable. For a stable equilibrium point, the system can return to it after a small perturbation, but for an unstable equilibrium point, the system will move away from it after a small perturbation. Those two situations are often illustrated with the ball-and-landscape metaphor, in which a stable equilibrium is like a ball in the lowest position of a valley, and an unstable equilibrium is like a ball at the highest position of a hill. Therefore, in the presence of noise, the system is more likely to remain close to stable equilibria and stay away from unstable equilibria. Note that, in some more complex cases, the system may not settle at an equilibrium point, but instead, move continuously along a certain trajectory, called a limit cycle. The tools introduced in this tutorial can also analyze limit cycles, yet a detailed discussion is beyond the scope of this tutorial. For details, we refer the interested readers to Strogatz (2018, Chapter 7).

The equilibria of a dynamic system can be altered by changing the *parameters*. Those parameters often define the strength of influence from one variable to another, or some intrinsic features of some variables’ dynamics. In principle, if we want to investigate the influence of parameters on the system’s dynamics, we can perform a series of phase space analyses for various parameter values (Fig. 3, Column C). However, this approach would be quite cumbersome in practice. A more concise method for summarizing how parameters influence the system’s dynamics would be useful. Looking at Column C, we can find that a minor change in the parameters only affects the *position* of equilibria, which is depicted in most cases. Only in some special cases, a change in the parameters can lead to changes in the stability of equilibria, the appearance of a new equilibrium point, or the disappearance of an existing equilibrium point. Those situations, where a small change in the parameters leads to a qualitative change in equilibrium points, are called *bifurcations*, and the method to evaluate whether a bifurcation is happening is called *bifurcation analysis*.

In a one-parameter bifurcation analysis, a plot is drawn to show the system’s position of the equilibrium points as a function of the parameter value (Fig. 3, Column D). The procedure of drawing such a plot consists of performing the phase plane analysis (Column C) many times, each time with a specific parameter value, and then putting the variable values of the corresponding equilibria states as dots in the one-parameter bifurcation plot on the corresponding parameter value (marked as red dotted lines on the first subplot of Column D). In the opposite direction, we can also infer the positions of the equilibrium points in the phase plane analysis (Column C) from slices of the one-parameter bifurcation analysis (the first subplot in Column D). In the first two subplots of Column D, we see two bifurcation points, at which a stable equilibrium and an unstable equilibrium point merged and disappeared. The system's behavior changes qualitatively when the parameter *a* crosses those two points. Between the two parameter points, the system has two stable equilibria; outside this range, it has only one equilibrium point. The bifurcation points may take different forms from the one shown in our example. Much mathematical work has been done in the 20th century to classify the types of bifurcation points. A more comprehensive overview of the types of bifurcation points is available in Thom (1975) or Abraham and Shaw (1992).

A dynamic model often contains multiple parameters. If we want to investigate how two parameters jointly influence the dynamics of the system, we can perform multiple one-parameter bifurcation analyses with various values of the second parameter, as in Column D of Fig. 3. However, most of the bifurcation analyses will only have small, quantitative differences, as is the case for the phase plane analyses. To examine how two parameters influence the system's behavior, we can perform a two-parameter bifurcation analysis. One-parameter bifurcation analysis can then be seen as taking slices of the two-parameter bifurcation, depicting the bifurcation points (compare Column D and Column E along the red dotted lines). The curve in a two-variable bifurcation analysis divides the parameter space into two regions. Each region has different numbers or types of equilibrium points. In the example of Column E, the region above the curve has two stable equilibria, whereas the region below the curve has only one stable equilibrium.

Conducting phase-plane analysis for more variables or bifurcation analysis for more parameters together is theoretically possible, but would require drawing plots in higher dimensions, which are difficult to comprehend (but see de Boer, 2024, for an application for 3D phase plane analysis). We therefore focus on the cases up to two dimensions in this article. To make the analysis readily available for psychologists, we introduce the R package *deBif*, developed by de Roos (2025)^3^. The deBif package provides an easy-to-use graphical interface that can be used to perform phase plane analysis and bifurcation analysis implemented by the functions phaseplane() and bifurcation(), respectively. The phaseplane() function can produce results like Columns A and B in Fig. 3, and the bifurcation() function can produce results like Columns C and D. Users can run those functions to evoke corresponding Shiny apps (Chang et al., 2024), and from there, perform various analyses interactively. The functions of the package, important parameters, and tabs are summarized in Table 3 and Table 4. We will illustrate the usage of the functions, the graphical interface, and specific analysis steps together with examples.

**Table 3:** Key functions of the deBif package, the tabs of the corresponding Shiny apps, and explanations

Function	Tab	Explanation
phaseplane()	Time plot	Show the simulated time series from the given starting point
	Nullclines	Show the nullclines
	Steady states	Show the steady states, and their stability, together with the nullclines
	Vector field	Show the nullclines, steady states, and the vector field (representing the direction of change) of different regions
	Trajectories	Show the simulation trajectory (in Time plot) on the phase plane, together with the nullclines and the steady states
	Portrait	Show multiple trajectories starting from different states, together with the nullclines and the steady states
bifurcation()	Time series	Show the simulated time series from the given starting point
	1 parameter bifurcation	Perform one-parameter bifurcation analysis (starting point required)^1^
	2 parameter bifurcation	Perform two-parameter bifurcation analysis (starting point required)

^1^ Researchers often need to supply a new starting point for those calculations, preferably from a steady state

**Table 4:** Key parameters for the functions in the deBif package

Parameter	Instruction
model	An R function that describes the dynamic system. The function should take three parameters, *t*, state, and parms, where *t* represents time, state is a named vector of all the variables, and parms is a named vector of all the parameters. The output of the function should be a vector of all the derivatives, in the order of the input variables. This model parameter is rather abstract. Readers may use the code we share as the starting point and modify it from there
parms	A named vector of all parameters. This parameter can be overwritten during the execution of the Shiny app
state	A named vector of the initial values of all the variables. This parameter can be overwritten during the execution of the Shiny app
…	There are other parameters to modify the graphic output in the Shiny app. See the help document of the functions for details

Many formal models contain more than two variables and more than two parameters. For those models, we still recommend that researchers focus on at most two variables and two parameters at the same time to enhance the interpretability of the model results. The treatment of other variables depends on the relative time scale of the system (Cui, Hasselman, et al., 2025b, 2025a; Hasselman, 2023). Variables evolving on faster timescales than the focus variables will reach their steady state quickly, so we can assume that their time derivative is zero, and therefore solve the fast variables as a function of slower variables to reduce the number of variables in the analysis (Bertram & Rubin, 2017; Okino & Mavrovouniotis, 1998). Variables that evolve much more slowly than the focus variables can be treated as parameters in the model rather than variables to investigate how changing their values affects the dynamics of the focus variables. We will show further illustrations with concrete examples in subsequent sections. These examples are based on the two nonlinear models introduced in the previous section, as nonlinear models can show more complex dynamic characteristics that are often the focus of the techniques we introduce here. For interested readers, we also provide an example with a linear model in Supplementary Materials A, as an easier starting point. In the examples below, we will show how to use code to evoke the GUI applications. For the first set of analyses, we also show detailed operations within the applications (see Supplementary Materials B for zoomed-in screenshots with better readability). The R code can also be obtained as an R script from https://osf.io/ym9vt/.

## Example 1: Analysis of the panic disorder model

The panic disorder model is specified by Eqs. (6–8), with the default parameters in Table 1. There are three core variables in the system: physical arousal (*A*), perceived threat (*PT*), and homeostatic feedback (*H*)*.*

## Analysis with *H* as a parameter

From both the default parameter values (Table 1) and the simulations (Fig. 2a), we can see that *H* changes at a much lower rate than the other two variables *A* and *T*. Hence, we first look at the faster time scale constituted by *A* and *T* and treat *H* as a parameter. The commands to revoke the phase plane analysis for the panic disorder model are as follows.
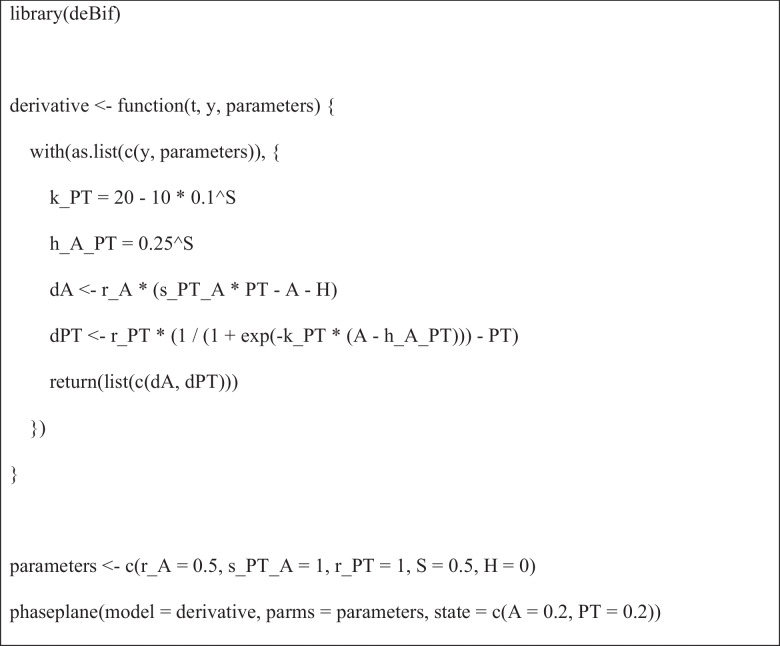


**Fig. 4:**
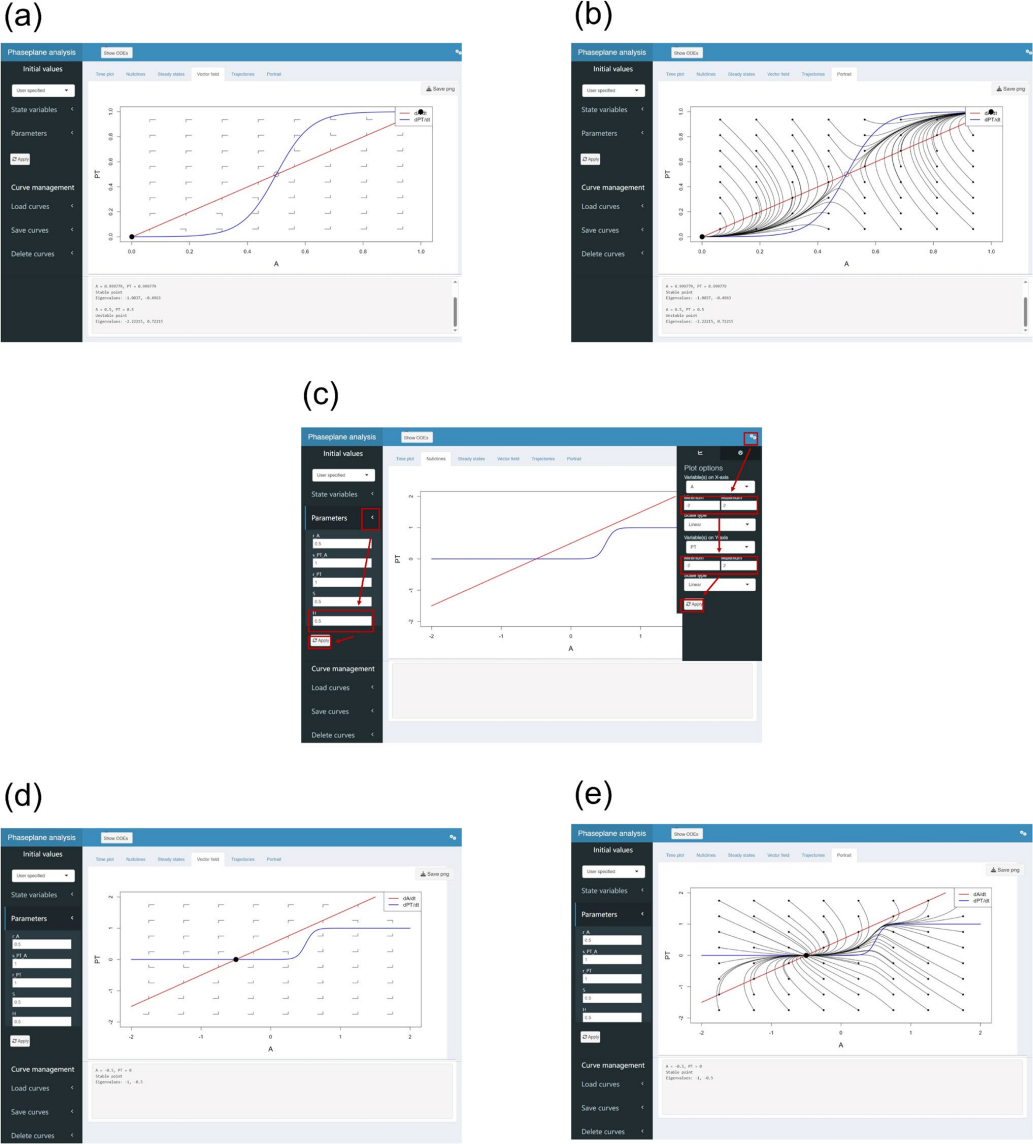
The phaseplane analysis app screenshots during the analysis of the panic disorder model, with *H* as a parameter. **(a)** The vector field tab for the default parameters; **(b)** the portrait tab for the default parameters; **(c)** the operation to change the default parameter values and the range of the variables; **(d)** the vector field tab for the adjusted parameters; **(e)** the portrait tab for the adjusted parameters. The vector field tab shows the nullclines (the *blue* and the *red lines*), the steady states and their stability (*solid points* for stable steady states and *hollow points* for unstable steady states), and the vector fields. To represent the vector fields, the application uses two components of the vectors instead of the vectors themselves. The portrait tab shows the nullclines, the equilibrium points, and their stability. The nullclines tab and the steady states tab can be used to show the nullclines and the steady states only (not shown here to save space)

**Fig. 5:**
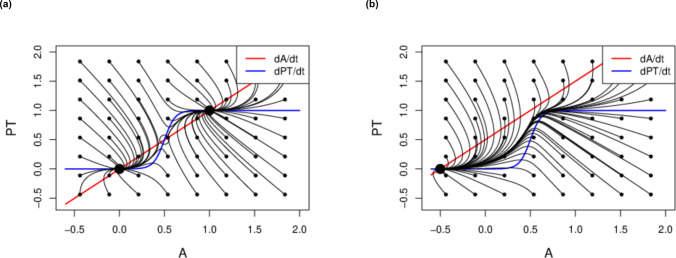
Phase plane analysis results of the panic disorder model, with *H* as a parameter. **(a)** *H* = 0; **(b)** *H* = 0.5. The plot shows the nullclines (the *blue* and the *red lines*), the steady states, their stability (*solid points* for stable steady states and *hollow points* for unstable steady states), and some trajectories

In the code above, the derivative function specifies the dynamic system, and the parameters are the default parameter values of the model, plus H=0. This value of H is rather arbitrarily chosen and will be altered in subsequent analyses. The function also requires a starting value of the system’s state, which can be an arbitrary meaningful value of the state variables. After running the code, we can see a pop-up window. Navigating on various tabs (see Fig. 4 or Supplementary Materials B), we can obtain the following results in Fig. 5.

We first investigate the results with the default parameter setting (Fig. 5a). The two nullclines represent the states where *A* and *PT* remain unchanged. The red line is the nullcline for *A*. For all the states to the lower right direction of this line, *A* decreases, and vice versa. The blue line represents the nullcline for *PT*. For all states to the upper left direction of this line, *PT* decreases. The two nullclines cross at three points, of which the first and the third are stable equilibria, and the middle one is an unstable equilibrium point. The two stable steady states represent the healthy and panic states of the system, respectively. From this analysis, it seems that the system can reside in either state, yet from the simulations (Fig. 2), we see that the system can only move to the panic state for a short time, and after that, quickly falls back to the healthy state. This needs to be explained by changing the value of *H*. After increasing *H* from 0 to 0.5 (and enlarging the display range of the variables accordingly, Fig. 5b), the dynamic features of *A* and *PT* change. From Fig. 5b, we can see that the nullcline of *A* (the red line) moves to the upper left direction. Now the two nullclines only intersect at one point, which is a healthy state, as both *A* and *PT* have a rather low value at this point. Therefore, the system can only gravitate towards a healthy state when *H* is high enough.

**Fig. 6:**
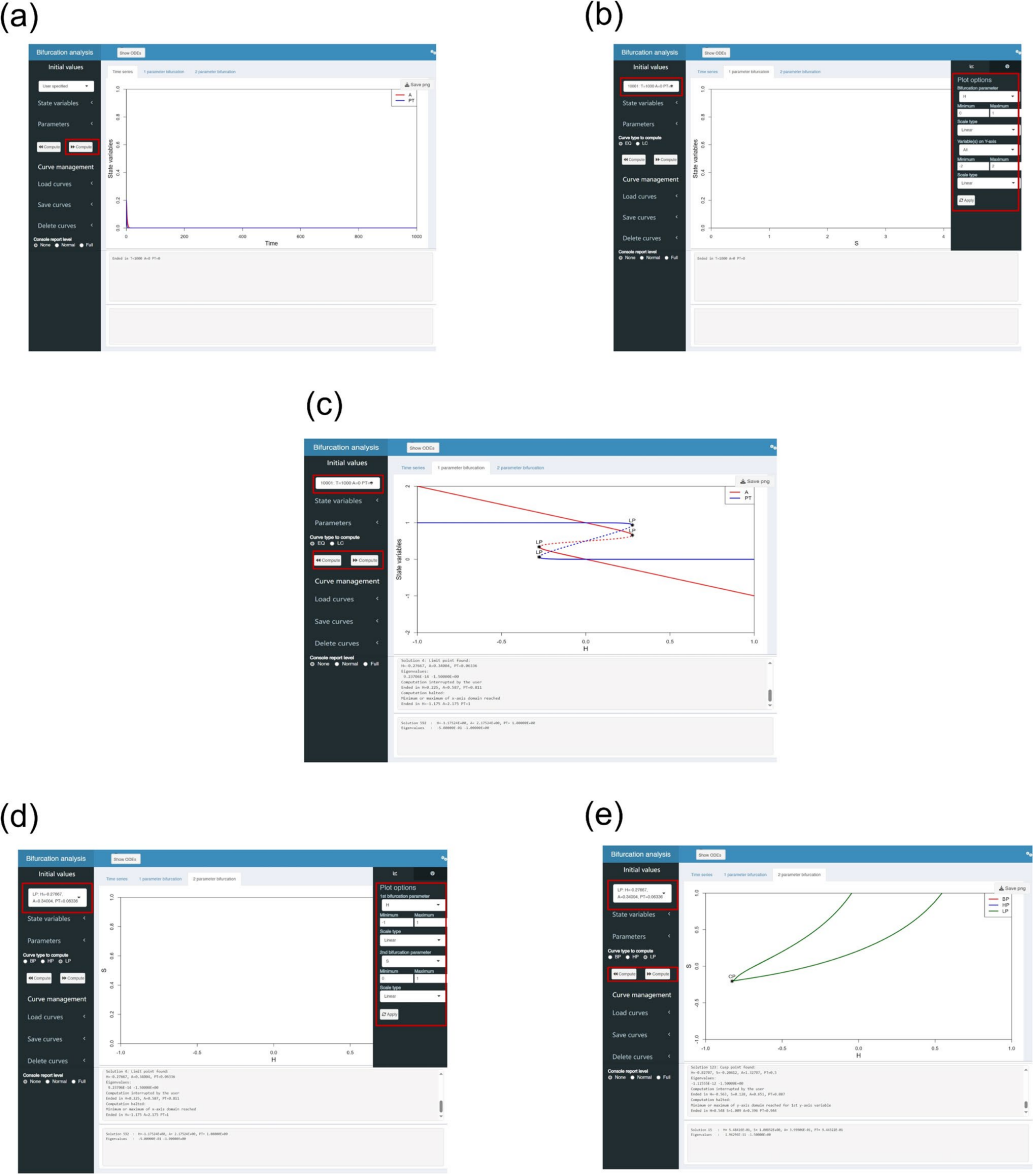
The bifurcation analysis app screenshots during the analysis of the panic disorder model, with *H* as a parameter. **(a)** The time series tab to perform a simulation. For bifurcation analysis, we need to use this tab to find one equilibrium point as the initial value for the subsequent analysis. Using arbitrary initial values will likely lead to errors. **(b)** The one-parameter bifurcation tab before performing the analysis. Use the panels marked in *red rectangles* to choose the initial value of calculation, the parameter of interest, and the ranges of parameters and variables. Those values can also be adjusted later. **(c)** The 1-parameter bifurcation tab with the analysis results. Use both the “calculate >>” and “calculate <<” buttons to let the program calculate in both directions and draw the full curve. If the maximum number of steps has been reached, you may need to choose the endpoint of the previous curve and recalculate to complete the curve. “LP” means limit points. *Solid lines* represent stable steady states and *dashed lines* represent unstable steady states. **(d)** The two-parameter bifurcation tab before performing the analysis. Use the panels marked in *red rectangles* to choose the initial value of calculation as one of the limit points in the one-parameter bifurcation step, the two parameters of interest, and the ranges of parameters and variables. Those values can also be adjusted later. **(e)** The two-parameter bifurcation tab with the analysis results. Again, use both the “calculate >>” and “calculate <<” buttons to let the program calculate in both directions and draw the full curve. If the maximum steps have been reached, you may need to choose the endpoint of the previous curve and calculate again to complete the curve, or start from another limit point. “CP” means cusp points. *Solid lines* represent stable steady states and *dashed lines* represent unstable steady states

**Fig. 7:**
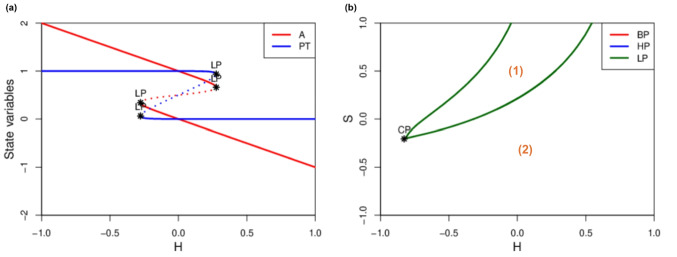
Bifurcation analysis results of the panic disorder model. **(a)** The one-parameter bifurcation analysis result, with *H* as the parameter. “LP” means limit points. *Solid lines* represent stable steady states, and *dashed lines* represent unstable steady states. **(b)** The two-parameter bifurcation result, with *H* and *S* as parameters. “CP” means cusp points, and “BP”, “HP”, and “LP” represent different types of bifurcation points, which we did not elaborate on in this tutorial. “HP” means Hopf bifurcation points (not present for this system), “BP” means branching points (not present for this system), and “LP” means limit points. The *points labeled with numbers* (e.g., 1, 2) were added by the authors for clarity and are marked in *orange*. These labels are not part of the raw software output


We can then perform the one-parameter bifurcation analysis by running the code below and using the GUI as shown in Fig. 6a–c or Supplementary Materials B. The results are shown in Fig. 7a. In this analysis, we can see that both variables *A* and *PT* have two stable states between two bifurcation points, where -0.28<H<0.28 (the specific coordinate values can be extracted from the Shiny application). If H<-0.28, the system only has the panic stable state, and if H>0.28, the system only has the healthy stable state. As *H* increases, the value of *A* for the stable states decreases both for the panic state and the healthy state, whereas the value of *PT* does not change much. Therefore, the bistability of the system only appears when homeostatic feedback is in a certain range, and the strength of homeostatic feedback also influences the level of physical arousal at the equilibrium states.

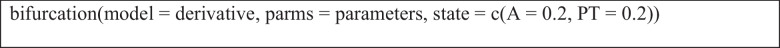


We can now include a second parameter in the analysis, *S* (Table 1). This parameter represents an important psychological construct, namely, arousal schema. It shows how much a person perceives high physical arousal as a sign of threat. In the original model by Robinaugh et al. (2024), arousal schema is a variable that changes at a much slower rate, and high arousal schema is seen as the key mechanism explaining panic disorder. We omitted the dynamic equation of *S* in our analysis as it takes an irregular form with if-else conditions and depends on the history of the system, thus cannot be analyzed with the methods introduced in this tutorial. Nevertheless, we can still use *S* in the two-parameter bifurcation analysis and see how this variable changes the stability of the steady states. We can conduct this analysis by using the GUI in Fig. 6d–e, and the result is shown in Fig. 7b.^4^ We can see that the two green curves together divide the parameter plane into two regions, (1) and (2). The two regions have different numbers of stable steady states. From the results of the one-parameter bifurcation analysis, we can infer that in the small triangle-like area (1), the system has two stable states, but in region (2), it has only one stable state. Specifically, when arousal schema is low, there is always only one stable state, but when arousal schema is high, the strength of the homeostatic feedback determines whether there is one or two stable states in the system and whether the stable state is a healthy state or a panic state. Thus, the stability feature of the system is dependent on the joint influence of both variables, and the panic disorder phenomenon can only be observed when the arousal schema is high enough and homeostatic feedback is moderate.

## Analysis with *H* as a variable

We then investigate the dynamics of *H*. To do so, we need to eliminate one of the fast variables to make the total number of variables two. This can be done by assuming that one fast variable is always at its equilibrium (i.e., the variable’s derivative is zero). The rationale for this treatment is that the fast variables approach their equilibrium much faster than *H*; hence, assuming that they are always at the equilibrium does not affect the results much (Bertram & Rubin, 2017; Okino & Mavrovouniotis, 1998). Here, we arbitrarily chose to solve *PT* (solving *A* would give similar results) from Eq. (7). Solving $\mathrm{d}PT/\mathrm{d}T=0$ results in:

12 $$PT=\frac{1}{1+\mathrm{exp}\left(-{k}_{PT}\left(A-{h}_{A,PT}\right)\right)}.$$

This equation can be used with the panic disorder model as presented in Eqs. 6–8 to run the phase plane analysis and the bifurcation analysis with the code below, and the GUI operations in Supplementary Materials B.
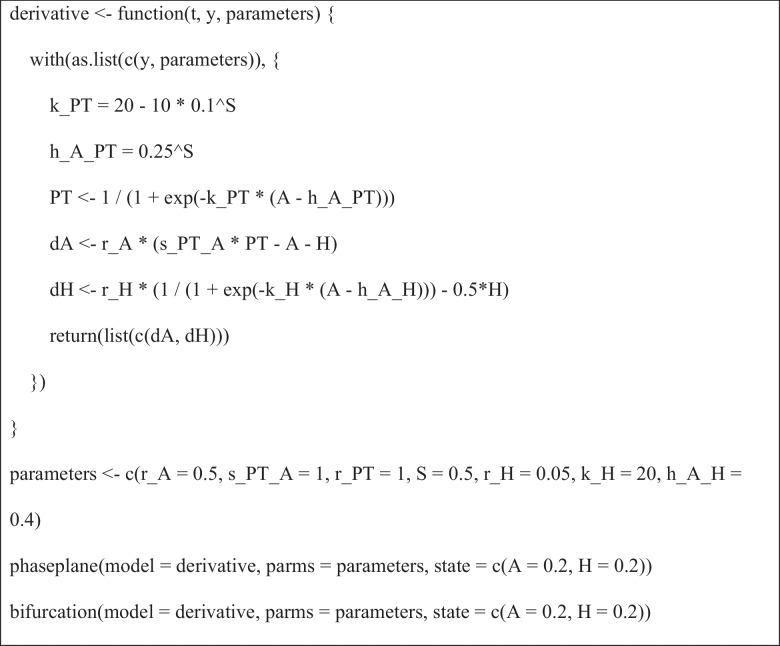


**Fig. 8:**
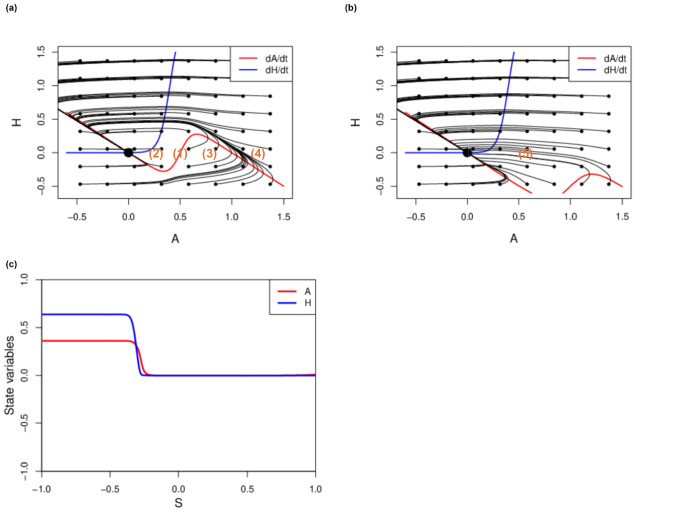
Phase plane analyses and bifurcation analyses results of the panic disorder model, with *H* as a variable. **(a)** phase plane analysis result using the default parameter (*S* = 0.5); **(b)** phase plane analysis result with *S* = 0; **(c)** one-parameter bifurcation analysis result. The *points labeled with numbers* (e.g., 1, 2) were added by the authors for clarity and are marked in *orange*. These labels are not part of the raw software output

The results are depicted in Fig. 8. We first look at the phase plane analysis results with arousal schema fixed at a moderate level, *S* = 0.5 (Fig. 8a). Although there is only one stable state in the system, represented by the large black dot close to (0,0), the system may move in opposite directions under noise: When *A* is above a certain threshold (1), it does not directly decrease towards the equilibrium of the system (the black dot). Rather, *A* first increases until its value reaches its nullcline (the red line) and then turns around and decreases again. This is only possible when the nullcline of *A* (the red line) has this particular curvilinear shape. If we look along the line of *H* = 0, there is first a region (2) where *A* decreases to make sure that the steady state is stable, then a region (3) where *A* increases to make it possible to show spikes, and then a region (4) where *A* decreases again to ensure *A* will not increase too much. Also, the change rate of *H* is low enough compared to *A* to make sure that the dynamic trajectories (the black curves in Fig. 8a) first almost go along the horizontal direction, so that *A* can show a clear spike.

We then use Fig. 8b to see why the system does not have panic attacks when *S* is fixed at 0. The results show that the nullcline of *A* (the red line) moves to the lower right direction, and compared to Fig. 5a, now the intersection point (1) disappears at (5). Therefore, even if there is noise driving *A* to higher values around (5), as the state of the system does not cross the nullcline of *A*, *A* will always tend to decrease, which means that there will be no panic spikes anymore (compare with the trajectories starting from (3)). From the bifurcation analysis result in Fig. 8c, we can see that as long as *S* is around the range between 0 to 1 (which is always the case in the complete model by Robinaugh et al., 2024), the position of the stable state hardly changes. Therefore, the level of physical arousal and the perceived threat of the healthy stable state will not change following the changes in *S*.

## Discussion of Example 1

In the analysis example shown above, we investigated the dynamics of the panic disorder model on two different time scales. For the first one, the fast time scale of *A* and *PT*, we found that both *H* and *S* can influence the stability of the system. When *S* is low, the system always has only one stable state, the healthy state (Fig. 7b). When *S* is higher, the stability of the system will depend on *H* (Fig. 7b). If *H* is at its default value (Fig. 5a), the system can have two stable states, one being healthy and the other being in panic. Therefore, with some noise, it is possible for the system to move from the healthy state to the panic state. But if *H* is higher (Fig. 5b), the panic state will become unstable again, and the system will fall back to the healthy state. The required *H* value to make the panic state unstable depends on *S*. The higher *S* is, the higher *H* must be to make the panic state disappear (Fig. 7b). The second part of the analysis focused on the slower time scale of *H*. We found that, because of the shape of the nullclines, if *A* is higher than a threshold (1), it will first increase before decreasing back to the healthy stable state, making panic spikes appear in the time series of *A*. However, in the long run, the system always tends to go back to the healthy state, as there is only one stable state in the system.

**Fig. 9:**
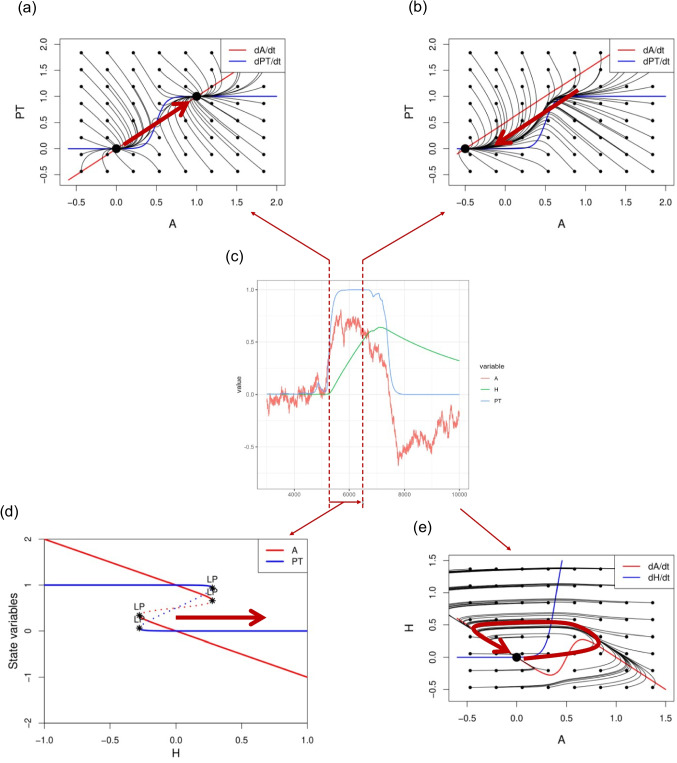
The relationship between the graphical analysis outputs and the simulated time series for the model of panic disorder. The *bold*, *red arrows* in panels **(a)**, **(b)**, **(d)**, and **(e)** correspond to various features of the time series in panel **(c)**. In the beginning part of the time series **(c)**, the system of *A* and *PT* is close to the phase plane analysis result in panel **(a)**. A bifurcation indicated in panel **(d)** happens during the period marked on panel **(c)**, making the nullclines of *A* and *PT* change from panel **(a)** to panel **(b)**. The entire process can also be understood in a slower time scale, illustrated by the phase plane analysis result in panel** (e)**

In Fig. 9, we provide an illustration to link the graphical analysis results back to the simulated time series. For Fig. 9c, we zoom into a segment of the time series shown in Fig. 2a, which represents a panic attack. The two panels above, which are the phase space analysis results for *H =* 0 (Fig. 9a) and *H* = 0.5 (Fig. 9b), correspond to the stability characteristics of *A* and *PT* with different *H* values. When *H* = 0, the subsystem of *A* and *PT* is bistable, thus noise is possible to drive the system from one stable equilibrium to another. However, later *H* increases, making the subsystem of *A* and *PT* monostable, forcing the system to go back to the only remaining equilibrium. This relationship between *H* and the stability of *A* and *PT* can also be found in the one-parameter bifurcation analysis, shown in Fig. 9d. Finally, in the phase space analysis on the slower time scale (Fig. 9e), we can see that after *A* increases from random noise, the system’s state has a tendency to go through a long detour before returning to the previous equilibrium, which corresponds to the phenomenon of panic attacks.

In summary, the key dynamic features giving rise to the spike-like behavior of the panic disorder model are the following: (1) The nullclines of *A* and *PT* may form one or two stable states depending on *H*. When *H* is high, two nullclines detach at the right side, making the stable panic state disappear. (2) The increase of *A* and/or *PT* can lead to a slow increase in *H*. We may change the specific function forms and still create similar behaviors in the system, as long as the key conditions are met. Robinaugh et al. (2021) tried several alternative forms of the functions of *A* and *PT* and suggested that only the combination of a linear form in the dynamic function of *A* (Eq. (6)) and the S-shape form in the dynamic function of *PT* (Eq. (7)) can produce behaviors like panic attacks. Based on the analysis above, however, we can conclude that this statement is likely not entirely correct. We infer that if both equations are S-shaped, the model can also show similar behavior. Indeed, we successfully formulated a model with both S-shaped function forms that matches the phenomenon of panic disorder, detailed in Supplementary Materials C. The graphical tools we propose here, thus, lead to a clearer explanation of why, or what exact mechanism, can lead to specific behaviors in the simulation results.

## Example 2: Analysis of the suicidal ideation model

To illustrate the generalizability of the proposed methods, we now move to the second example, the suicidal ideation model by Wang et al. (2023). The model contains three core variables: aversive internal states (*A*), urge to escape (*U*), and suicidal thoughts (*T*). The model is specified by Eqs. 9–11, with the default parameter values shown in Table 2.

The key feature of the model is that, although the aversive internal states and urge to escape have many peaks and fluctuations, suicidal thoughts are highly zero-inflated and only have a few peaks (Wang et al., 2023; also see Fig. 2b). We now investigate why the system has this feature. As the system has three variables with similar time scales (Eqs. 9–11), we arbitrarily solve for the variable *U* by assuming $\mathrm{d}U/\mathrm{d}t=0$. From Eq. 10, we have:

13 $$U=\frac{{b}_{3}A}{{c}_{3}}.$$

**Fig. 10:**
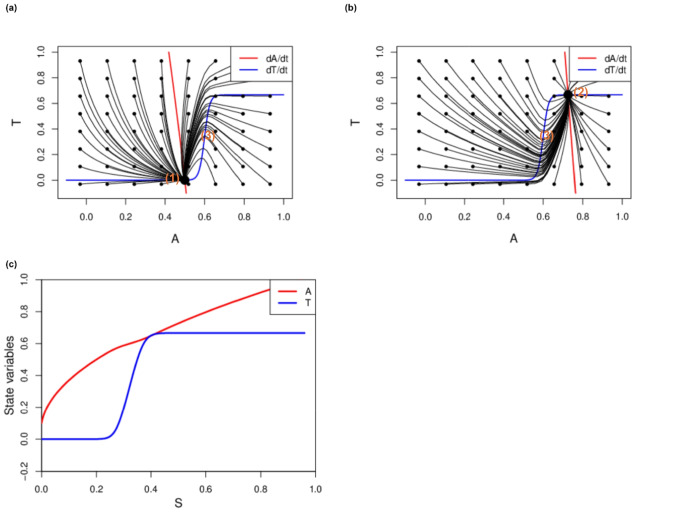
The phase plane analysis and bifurcation analysis screenshots during the analysis of the suicidal ideation model. **(a)** is the phase plane analysis result with *S* = 0.2; **(b)** is the phase plane analysis result with *S* = 0.5; **(c)** is the one-parameter bifurcation analysis result with *S* as the parameter. The *points labeled with numbers* (e.g., 1, 2) were added by the authors for clarity and are marked in *orange*. These labels are not part of the raw software output

**Fig. 11:**
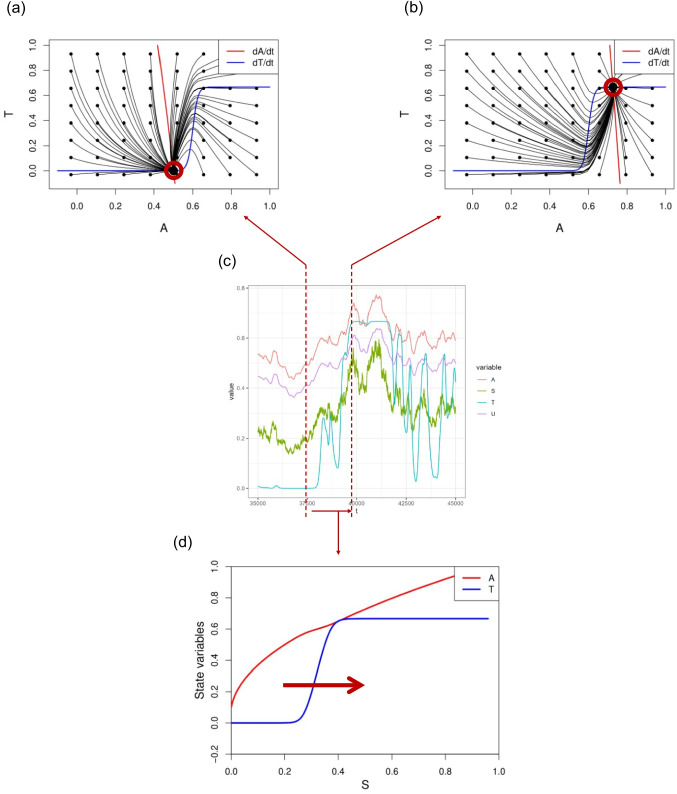
The relationship between the graphical analysis outputs and the simulated time series for the model of suicidal ideations. The *bold*, *red circles* and *arrows* in panels **(a)**, **(b)**, and **(d)** correspond to various features of the time series in panel **(c)**. In the beginning part of the time series **(c)**, the system of *A* and *T* is close to the phase plane analysis result in panel **(a)**. A change of the equilibrium indicated in panel **(d)** happens during the period marked on panel **(c)**, making the nullclines of *A* and *T* change from panel **(a)** to panel **(b)**


To investigate how different variables of the system react to random stressors, we use the variable representing the external stressor, *S*, as the model parameter in our analysis. We can conduct the analysis with the code below and the GUI operations in Supplementary Materials B. The results are shown in Fig. 10. We can see that the system always has a single stable point, but at different locations. As *S* increases from *S* = 0.2 in Fig. 10a to S = 0.5 in Fig. 10b, the nullcline of *A* (the red line) moves to the upper right direction, making its intersection with the nullcline of *T* (the blue line) move from (1) to (2). The nullcline of *T* is S-shaped, so that at the beginning, the intersection does not change much in the *T* axis (Fig. 10a). After a threshold (3), however, the intersection of the nullclines moves to the second half of *T*’s nullcline, so the *T* value of the stable point suddenly increases (Fig. 10b). We can see this trend more clearly from the bifurcation analysis in Fig. 10c. Here, as *S* increases, *A* increases smoothly, but *T* increases abruptly. Therefore, if *S* randomly fluctuates, we can observe that *A* follows closer with *S*, whereas *T* mostly stays around zero, with occasional large spikes. This follows the simulation results shown earlier (Fig. 2b). Again, in Fig. 11, we show another illustration that emphasizes the relationship between the simulated time series and the results from phase plane analyses and a bifurcation analysis.

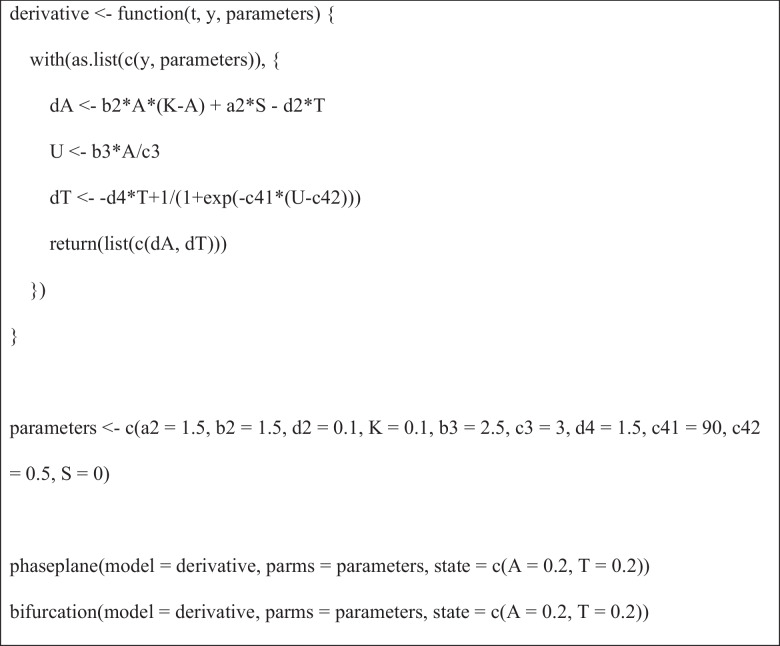


In summary, the difference between the dynamics of *A* and *T* can mainly be explained by the shape of their nullclines. The nullcline of *A* is close to a straight line with a large, negative derivative and moves smoothly in the right direction, and the nullcline of *T* is close to a S-shaped curve that does not change with *S*. Again, in Supplementary Materials C, we formulated an alternative model in which the dynamic function of *A* takes a linear form instead of a quadratic form, while retaining the relationship between the nullclines. The characteristics of this model closely resemble those of the original model of suicidal ideation, yet the alternative model has a simpler form. Therefore, using the analysis methods presented in this tutorial, we can see that the quadratic expression in the original model (Wang et al., 2023) is not necessary to produce the phenomenon of interest.

## General discussion

In this tutorial, we introduced two important graphical tools for dynamic systems, namely the phase plane and bifurcation analysis. We explained the meaning of several important plots of the analyses, the related code, and the procedure, and we demonstrated the analysis with two specific formal dynamic models in the psychopathology domain. For the panic disorder model, we found that the key feature of the system, namely the spikes of panic attacks, comes from fast, potentially bistable dynamics between physical arousal (*A*) and perceived threat (*PT*), and a slower variable, homeostatic feedback (*H*), that controls the dynamics of *A* and *PT*. When *H* hovers around the baseline level, the system of *A* and *PT* is bistable, so it may go close to the panic state under noise. However, this will lead to an increase in *H*, which makes the system of *A* and *PT* monostable, leaving only the healthy state, so that after a short period of time in the panic state, the system will quickly return to the healthy state. The variable that is even slower, *S*, controls the required level of *H* to make *A* and *PT* monostable, thus controlling whether the system is likely to show panic attacks. For the suicidal ideation model, we found that the different reactivity of aversive internal states (*A*) and suicidal thoughts (*T*) towards external stressors comes from the shape of their nullclines. The nullcline of *A* is close to a straight line and moves smoothly with increasing external stressors (*S*), whereas the nullcline of *T* is an S-shaped curve. When* S* increases, the intersection point moves along the nullcline of *T*, leading to a smooth change in *A* and an abrupt change in *T*.

For both models, we proposed alternative forms of dynamic equations that are different from the original specifications but still meet the key dynamic features we found. This underscores the importance of the graphical tools we introduced in investigating the underlying dynamics producing a certain psychological phenomenon. Both adapted models showed similar behaviors compared to the original models. Therefore, in contrast to previous arguments by some researchers (e.g., Robinaugh et al., 2021), the alignment between simulation results and real-life observations does not necessarily provide robust support for the exact function form used in formal models. This may render further deductions from those models (e.g., in silico experiments of treatments, Ryan et al., 2025) less reliable. Instead, we argue that greater emphasis should be placed on the key dynamic features of the model.

The aim of introducing those graphical methods to the realm of psychology is to gain a better understanding of the mechanisms underlying certain dynamics. In other words, we do not only want to have computational models that behave similarly to real-life systems, but we also want to understand which characteristics of the dynamic functions can give rise to a specific phenomenon, hence feature in the time series. Formal dynamic models are often defined with a set of functions and parameters. If the simulation results show the key features of the dynamic model, we can only infer that this specific combination of function forms and parameter values may be a reasonable candidate model for the phenomenon of interest. The amount of information we can gain from a single instance, though, is rather little, as there are many other possibilities of function forms and parameters that may lead to similar results. Optimally, we at least want to have a group of models featured by a set of similar characteristics that may give similar results. Based on this, future research may also go further to develop a more comprehensive classification system of dynamic function forms and the dynamic features of their combinations. In Supplementary Materials D, we provide some examples with dynamic functions in quadratic and cubic forms, which may serve as a starting point for expanding and classifying the building blocks for future dynamic models. Many researchers claim that the benefits of using formal models are to reduce the ambiguity of verbal descriptions and make the theory more specific (Borsboom et al., 2021; Fried, 2020; Robinaugh et al., 2021), but relying on a single simulation model also has the risk of seeing the trees instead of the forest. By focusing on the characteristics of formal dynamic models instead of specific instances of formal models, we can balance specificity and generalizability and make the results more interpretable.

Analyzing the model at the level of dynamic features also enables model comparison across fields. Several types of models are known in other fields, which may describe very different phenomena but share certain dynamic features. For instance, excitable models in biology, originating from the research on neural activation, describe how a system may leave its equilibrium and go towards another direction, showing a spike in the signal, before returning to the original equilibrium point (Edelstein-Keshet, 2005; Murray, 1989), are very similar to the panic disorder model. Thus, the conditions in excitable models may also apply to formal models of panic disorder. Alternatively, if we want to develop a new model for another type of psychological phenomenon and are aware of models in other fields that exhibit similar behaviors, we can use those models as a starting point and adapt them to build psychological models. For example, the Hopf bifurcation, which describes how a dynamic system transitions from a steady state to a limit cycle and exhibits oscillatory behavior, may be a useful starting point to build a model of bipolar disorder and investigate how a client changes from having a balanced mood to experiencing alternating periods of manic and depressive phases.

The field of dynamic modeling in psychology (and the domain of psychopathology specifically) is still in its infancy. Pioneering work in this field (e.g., the models we used as examples by Robinaugh et al., 2024; Wang et al., 2023; and other models described by Burger et al., 2020; Schöller et al., 2018; van Dongen et al., 2025) often involves a large number of variables or constructs, while the resulting dynamics tend to be relatively simple, with only a few key features of interest. At the same time, it is unclear whether these features arise from the complex interactions among elements or are primarily driven by one or two functions specified by the researcher. Having many elements and complex dynamic functions is not necessarily a problem. However, its level of complexity should be justified by the complexity of the model’s outcomes.

Here, it is good to refer to the work by Levins (1966), who has defined three purposes of dynamic modeling: generality, realism, and precision. In reality, those three goals are hard to achieve within the same model. Generality emphasizes the basic understanding of the system’s core mechanism. A generalizable mechanism can be transferred to many similar situations, but it goes at the cost of ignoring less important elements, and the quantitative predictions may not be precise. Realism prioritizes resemblance between the model and real-life situations, for example, the number and nature of the elements involved and the forms of interaction. Practically, this may make the system very complex and hence difficult to disentangle the basic mechanisms that give rise to the phenomenon. It will also make the model less generalizable to other, similar situations, and its predictive power may be weaker than that of simpler models due to the difficulty of estimating all parameters in such a complex model. Lastly, a model can also aim to achieve high prediction precision. Machine learning models, for example, would be suitable for this goal, but it is worth noting that prioritizing prediction precision often comes at the cost of the model's generalizability and realism.

The field of psychology currently lacks a basic understanding of the core dynamical mechanisms underlying many psychological phenomena. Striving for high realism by constructing complicated models involving many variables might therefore be premature. Given that most simulation outputs are only qualitatively compared to empirical data, aiming for high precision also seems less relevant. We therefore advocate prioritizing the generalizability of the underlying mechanisms of the system by building simpler models with fewer core variables, facilitating investigation and interpretation. The introduced methods greatly contribute to this endeavor by enabling a more systematic investigation of formal models, thereby expanding the potential of formal dynamic models to advance psychological theories.

## Supplementary Information

Below is the link to the electronic supplementary material.

Supplementary file1 (PDF 4.07 MB)

## Data Availability

The data used in the current article can be generated with the code described in the Code availability statement.

## Footnotes

1 This work is still in the preprint stage, and the exact form of functions may be subject to future revisions. The preprint version we based on is the version edited on June 20, 2024, available at https://osf.io/download/2bptk/?version=2.

2 Strictly speaking, this is the definition of 2D phase space, which is rather easy to interpret and informative. Therefore, in the remaining part of this article, we use the term phase space to represent 2D phase space by default.

3 The package also includes a vignette with detailed technical guidance, available at https://cran.r-project.org/web/packages/deBif/vignettes/deBif-manual.pdf.

4 Note that applying two-parameter bifurcation analysis to complex models using automatic software, such as the deBif package, can encounter convergence issues. Results may also be highly sensitive to initial conditions and numerical settings. While these problems did not arise in the relatively simple model we analyzed, readers should be aware of them and consider alternative approaches (such as analytical bifurcation methods or multiple one-parameter analyses) if errors occur.

## Code availability

All code to reproduce the results in the current article is available on an OSF repository (https://osf.io/ym9vt/) under the GPL-3 license. The detailed operations to obtain the results from Shiny apps are described in the Supplementary Materials.

## Declarations

### Open practices statement

All code to reproduce the results in the current article is available on an OSF repository (https://osf.io/ym9vt/) under the GPL-3 license. The detailed operations to obtain the results from Shiny apps are described in the Supplementary Materials.
