# Supplementary material for: Analyzing multidimensional formal dynamic models in psychology: A tutorial using graphical tools
Source: Behav Res Methods. 2026 Jun 22;58(8):206. doi: 10.3758/s13428-026-03077-y (PMC13287130; doi:10.3758/s13428-026-03077-y)
Supplement: Supplementary file 1 — Supplementary file1 (PDF 4.07 MB) [file 13428_2026_3077_MOESM1_ESM.docx]

**Supplementary Materials:**

The supplementary materials associated with this manuscript are available at <https://osf.io/ym9vt/>.
